# Role of Host Cell Secretory Machinery in Zika Virus Life Cycle

**DOI:** 10.3390/v10100559

**Published:** 2018-10-15

**Authors:** Garrett Sager, Samuel Gabaglio, Elizabeth Sztul, George A. Belov

**Affiliations:** 1 Department of Cell, Developmental and Integrative Biology, University of Alabama at Birmingham, Birmingham, AL 35294, USA; gsager56@uab.edu; 2 Department of Veterinary Medicine, Virginia-Maryland Regional College of Veterinary Medicine, University of Maryland, College Park, MD 20742, USA; samuelgv@terpmail.umd.edu

**Keywords:** Zika virus, flaviviruses, virion maturation, secretory pathway, membrane trafficking

## Abstract

The high human cost of Zika virus infections and the rapid establishment of virus circulation in novel areas, including the United States, present an urgent need for countermeasures against this emerging threat. The development of an effective vaccine against Zika virus may be problematic because of the cross reactivity of the antibodies with other flaviviruses leading to antibody-dependent enhancement of infection. Moreover, rapidly replicating positive strand RNA viruses, including Zika virus, generate large spectrum of mutant genomes (quasi species) every replication round, allowing rapid selection of variants resistant to drugs targeting virus-specific proteins. On the other hand, viruses are ultimate cellular parasites and rely on the host metabolism for every step of their life cycle, thus presenting an opportunity to manipulate host processes as an alternative approach to suppress virus replication and spread. Zika and other flaviviruses critically depend on the cellular secretory pathway, which transfers proteins and membranes from the ER through the Golgi to the plasma membrane, for virion assembly, maturation and release. In this review, we summarize the current knowledge of interactions of Zika and similar arthropod-borne flaviviruses with the cellular secretory machinery with a special emphasis on virus-specific changes of the secretory pathway. Identification of the regulatory networks and effector proteins required to accommodate the trafficking of virions, which represent a highly unusual cargo for the secretory pathway, may open an attractive and virtually untapped reservoir of alternative targets for the development of superior anti-viral drugs.

## 1. Introduction

Zika virus is a positive strand RNA ((+)RNA) virus belonging to a group of mosquito-borne flaviviruses that includes Dengue viruses, West Nile virus, yellow fever virus, Japanese encephalitis virus and a growing number of other less-known viruses with potential as human pathogens [1]. Zika virus was discovered in rhesus monkeys in Uganda in 1947 and, although serological surveys demonstrated wide distribution of Zika infection in human populations throughout Africa, India and South-East Asia, until recently the virus was considered an obscure tropical pathogen of little importance to public health [2,3,4,5]. The virus emerged in the spotlight after major outbreaks in the South Pacific in 2007, 2013–2014 and the most recent one in Brazil and other South American countries [6,7,8,9]. Among the most severe consequences of the introduction of the virus in the previously Zika-naïve populations were increased incidence of Guillain-Barré syndrome, a devastating autoimmune disorder targeting the nervous system and congenital defects including microcephaly [10,11]. The rapid spread of the virus, which effectively established global presence in the tropical regions, similar to the distribution of Dengue viruses and the high human cost of infections prompted the WHO to declare Zika a public health emergency of international concern in 2016. 

Zika virus persists in enzootic cycle between primates (including humans) and mosquitoes of *Aedes* genus, especially *Aedes albopictus* and has a high potential for establishing circulation in other mammalian and mosquito species (reviewed in Reference [12]). In contrast to other related mosquito-borne flaviviruses, Zika virus seems to be unique in its capability to persist for months in immune-privileged sites, such as eyes and testes and to be transmitted sexually [13,14]. The capacity of the virus to persist in immune-privileged sites may represent a significant hurdle in designing an effective vaccine. Moreover, implementation of an anti-Zika vaccine may be problematic in the areas where Zika virus co-circulates with Dengue viruses since cross-reactivity of Dengue and Zika virus antibodies has been demonstrated in cell culture and animal studies to lead to antibody-dependent mutual enhancement of infection, underscoring the necessity to develop alternative approaches against this emerging virus [15,16,17,18,19].

Rapidly replicating (+)RNA viruses, including mosquito-borne flaviviruses, are notorious for their ability to develop resistance to compounds targeting viral proteins [20,21]. On the other hand, viruses rely on cellular metabolism for every step of their life cycle, providing an opportunity to control infections by manipulating host rather than viral factors. Cellular proteins do not change, thus targeting cellular factors critical for infection instead of easily adaptable viral proteins likely poses a higher barrier for development of resistance. Moreover, even distantly related viruses rely on highly conserved replication mechanisms and likely share the requirements for the same cellular factors, thus providing an opportunity for developing broadly effective therapeutics with high barrier of resistance [22]. 

Zika and related flaviviruses critically depend on the cellular secretory pathway for virion formation, maturation and release, as well as for secretion of the viral protein NS1, an important modulator of host immunity. Such dependence may represent an especially vulnerable step of the viral life cycle. Trafficking of the virions requires extensive modifications of the secretory pathway to accommodate the large particulate cargo. Thus, the membrane landscape of infected cells should significantly differ from that in uninfected ones, providing an opportunity to develop interventions specifically targeting cells supporting active virus replication. 

Zika infection in a mammalian host proceeds through sequential engagement of different types of cells. The virus from the original inoculum delivered in a mosquito bite infects nearby skin cells, such as skin fibroblasts and keratinocytes and is eventually picked up by skin-resident dendritic cells (Langerhans cells) that deliver the virus to the draining lymph nodes [23]. Infection of monocytes and macrophages infiltrating the lymph nodes leads to mounting viremia, necessary for subsequent transmission of the virus to new mosquito vectors during blood meal. Circulation of infected monocytes in the blood stream also allows the virus to reach other sites in the body, including those important for the development of Zika-specific pathologies and persistence, such as placenta and testes [24,25]. Thus, for successful sustained infection, the virus has to be able to navigate different cell-specific secretory pathway landscapes. Moreover, since the viral transmission cycle also requires replication and virion production in a mosquito vector, the virus has to maintain the ability to also engage the arthropod secretory pathway. This implies that the virus likely targets similar, highly evolutionarily conserved elements controlling the functionality of the secretory pathway in diverse organisms. Here we take a cellular biology-focused, rather than the usual virus-centric approach, to summarize the current understanding of the engagement of the cellular secretory machinery in Zika (and related flaviviruses)-infected cells and seek to highlight the areas where our knowledge is particularly scarce. The detailed understanding of this critical virus-cell interaction could open novel avenues for the development of better infection control strategies. We focus our discussion mainly on virion trafficking, as the mechanisms involved in secretion of the flavivirus protein NS1 have been recently reviewed in References [26,27]. Table 1 provides a succinct summary of the current state of knowledge of the involvement of cellular secretory pathway factors in the flavivirus life cycle. 

## 2. Zika Virus Genome Organization and Replication Cycle

**Figure 1:**
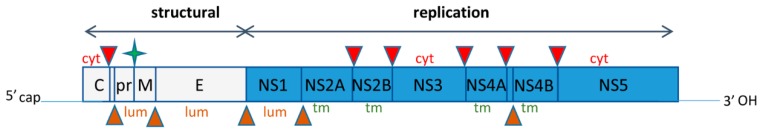
Scheme of Zika virus genome. The distribution of individual peptides and cleavage sites relative to the ER membrane are indicated. Cyt—cytoplasmic side, lum, -luminal side, tm—transmembrane. Red triangles designate cleavages performed by the viral protease complex NS2B-NS3 on the cytoplasmic side of the ER; brown triangles indicate cleavages performed by cellular proteases inside the ER lumen; green star shows final maturation cleavage of M glycoprotein performed by the Golgi-resident protease furin.

Zika virus genome RNA of ~10,500 nt has a 5′ cap structure but lacks a poly-A tail and codes for one polyprotein that contains three structural peptides (capsid protein (C), envelope (E) and membrane (prM/M) glycoproteins) in its N-terminus, followed by non-structural proteins responsible for RNA replication and evasion of the host anti-viral response (NS1-NS5A) (Figure 1). Translation of the flavivirus RNA is likely initiated in the cytosol immediately upon release from the virion. The N-terminal part of the nascent polyprotein contains an ER-localization signal that promotes rapid association of ribosomes translating the viral RNA with the ER membranes. This results in co-translational insertion of the growing polyprotein into the ER, leading to a complex distribution of the individual protein domains: PrM, E, NS1 and some extended stretches of NS2A, NS4A and NS4B are located intraluminally, while C, NS3 and NS5 are facing the cytoplasmic side of the ER, with several transmembrane sequences present in NS2A, NS2B and NS4B traversing the ER membrane bilayer (reviewed in Reference [28]). 

The polyprotein is processed into individual peptides by virally-encoded protease complex NS2B-NS3 that cleaves bonds exposed on the cytoplasmic side of the ER, as well as cellular proteases including signal peptidase that cleave bonds located in the lumen of the ER (Figure 1). The final cleavage of the glycoprotein prM to generate its mature form M required for virion infectivity is performed by the Golgi-resident protease furin [29]. The non-structural proteins form replication complexes on modified ER membranes and first transcribe genomic (+)RNA into a minus strand RNA that serves as a template for producing multiple copies of progeny (+)RNAs. The newly-synthesized (+)RNAs can be either recruited for further rounds of translation/replication or incorporated into virions, which also initiate their assembly on ER membranes. Assembled virions enter and then travel through the secretory pathway, undergoing maturation along the way, until their final release into the extracellular space. 

## 3. Overview of the Secretory Pathway

The cellular secretory pathway is a complex trafficking network mediating movement of proteins from the ER to other organelles, including the plasma membrane, where they may remain associated with the cell surface, or are secreted into the extracellular space. The secretory pathway operates in all eukaryotic cells and is an essential housekeeping process facilitated by a set of highly conserved molecules [30,31,32,33]. However, in multicellular organisms the secretory pathway is further specialized to perform cell-specific functions and the trafficking capacity and the adaptation to transport specific cargos are significantly different among different cell types. Such adaptation is supported by the extensive evolution of additional trafficking components, most often by gene duplication and neo-functionalization of pre-existing key components [34]. Thus, while the main steps of the secretory pathway and the key regulatory networks controlling them are conserved from yeast to humans, the human genome encodes several orthologs of yeast proteins, specialized for handling different types of cargos. 

**Figure 2:**
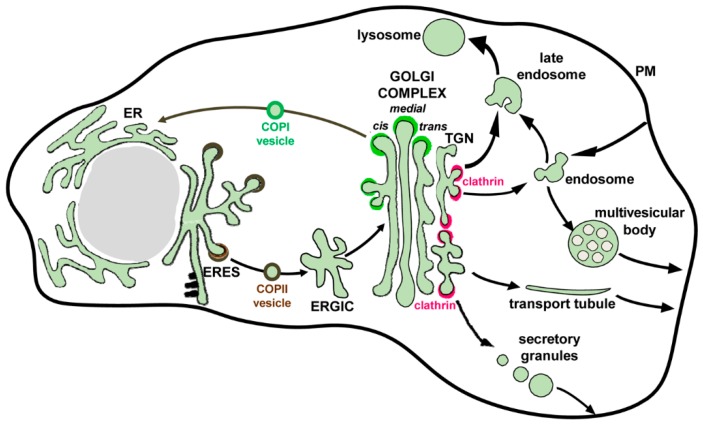
Compartments of the secretory pathway. Transport steps are indicated by arrows. Secretory cargos are synthesized in the ribosome-studded ER, exit the ER at ERES in COPII-coated (brown) vesicles and are transported to the ER-Golgi compartment (ERGIC) and then to the Golgi. After passage through the Golgi complex in the cis-to-trans direction, cargos are packaged at the TGN for delivery to the PM, early and late endosomes and in some cells to secretory granules. Sorting into endosomal compartments and secretory granules is mediated by clathrin-coated (red) vesicles. Transport to the PM is mediated by transport tubules. A COPI-mediated (green) recycling pathway retrieves escaped proteins from the ERGIC and the Golgi and returns them to the ER. Multivesicular bodies form by invaginations of endosomal membrane into its lumen and can fuse with the PM to release their content of exosomal vesicles.

All the proteins to be secreted are synthesized by ER-associated ribosomes and are either partially (transmembrane proteins) or completely (soluble proteins) translocated across the ER membrane into the lumen of this extensive tubular organelle (Figure 2). While in the ER, the newly-synthetized proteins interact with ER-resident chaperones that facilitate proper folding of the proteins and undergo initial glycosylation modifications. The time spent at the ER is an important element of protein quality control, which is necessary to select properly folded proteins to be moved further along the secretory pathway and to remove defective/misfolded proteins for degradation [35]. Protein exit from the ER proceeds at the ER exit sites (ERES), specialized ER domains where proteins to be secreted are concentrated and packaged into COPII transport vesicles. The proteins destined for secretion contain specific signal sequences that mediate their interaction with cargo adaptor proteins that ensure sorting into COPII-coated vesicles, thus segregating secretory proteins from ER-resident factors [36,37]. COPII vesicles deliver their cargo to the next transport station, the ER-Golgi Intermediate Compartment (ERGIC) and from there, the proteins are transported to the Golgi. The anterograde flow of membranes and cargo is balanced by the efficient recycling of membrane and escaped ER proteins from the Golgi back to the ER that is maintained by COPI-coated vesicles, thus supporting the dynamic steady-state equilibrium between these organelles [38]. 

The Golgi is a stack of membranous cisternae arranged in a cis, medial and trans orientation, with the cis side facing the ER and the trans side facing the plasma membrane (Figure 2). Two models have been proposed for the movement of cargo proteins through the Golgi stack. One states that the proteins are transferred between the Golgi cisternae via vesicles, similar to the trafficking between the ER and the ERGIC. In this view, the Golgi cisternae represent dynamic but relatively stable entities maintaining their relative positions for a prolonged period of time. The other model posits that movement through the Golgi involves the whole cisternae, so that they form *de novo* at the cis side by incoming ERGIC compartments, while the cisternae at the trans side disappear by transforming into the extensive tubular trans Golgi network (TGN). TGN then generates specific carriers to move proteins to their final destinations, including the plasma membrane [39,40]. The models are not mutually exclusive and there is evidence that different cargoes are primarily transported by one or the other mechanism. It is also possible that in different cell types, one or the other model may function preferentially. During their passage through the Golgi, the proteins undergo final glycosylation modifications and many are proteolytically processed by the Golgi-resident enzymatic machinery [41]. The delivery of secretory proteins to the plasma membrane can proceed through the fusion of tubular transport tubules directly emanating from the TGN, through the release of TGN-derived secretory granules, or through the fusion of post-TGN endosomal compartments such as multivesicular bodies (Figure 2). 

## 4. The Secretory Pathway in Zika Virus Infection: ER Modifications

The ER undergoes massive remodelling in cells infected with Zika and other arthropod-borne flaviviruses. The infection-induced changes of ER architecture likely disturb normal balance of the secretory trafficking factors and condition the cell for subsequent virion secretion. EM images of Zika-infected cells reveal infection-specific modifications of the ER membranes, generating spatially distinct domains that support specific processes of the virus replication cycle. 

The RNA translation and initial polyprotein processing are believed to be associated with so-called convoluted membranes (CM) [42,43,44,45] derived from local proliferations of the ER membrane, which form extensive folds and are often arranged in paracrystalline arrays [43,44,46,47]. The mechanism underlying the development of CM is not clear but it is likely to be at least partially dependent on activation of new lipid synthesis, required to support ER membrane growth. Indeed, it was shown that Zika-related arthropod-borne flaviviruses, such as West Nile and Dengue viruses, actively recruit cellular fatty acid synthase, an enzyme that provides long chain fatty acids necessary for membrane lipid synthesis, to the replication sites [48,49,50]. Interestingly, the characteristic CM are detected only in mammalian cells infected with Dengue virus but not in a mosquito-derived C6/36 cell line, indicating significant host-specific differences in the development of infection [45]. 

Viral RNA replication proceeds in the invaginations of the rough ER membrane into the ER lumen connected to the cytoplasm by a narrow neck [43,51,52]. Different strains of the Zika virus generate replication invaginations of different size in the same cell type and infection with the same virus induces invagination of different size in different cell types, suggesting the coordinate involvement of both host and viral factors in their development [44]. Interestingly, the diameter of the neck connecting the invaginations to the cytoplasm is similar regardless of the cell type or the virus strain and was even comparable in cells infected with either Zika or Dengue viruses, suggesting a conserved viral and/or cellular machinery involved in their formation [44]. Individual replication invaginations are often clustered together to form so-called vesicle packets, whose formation and morphology depends on the recruitment of a cellular protein reticulon 3.1A, a host factor involved in the maintenance of ER structure [43,51,52,53]. The exact role that reticulon 3.1A plays in packets formation is unclear but it may function to corral viral and host proteins within a limited area by restricting lateral diffusion within the plane of the ER membrane. The 3D electron tomography images suggest that CM and vesicle packets may be connected by one continuous sheet of membrane derived from the ER [43] but how such complex architecture is achieved and maintained remains to be determined. Expression of only the viral integral membrane protein NS4A is sufficient to induce invaginations morphologically similar to those observed in infected cells, although NS4B could also contribute to their development [54,55,56]. Still, the mechanism of how NS4A alters ER homeostasis and which host factors are required to support the development of invaginations is unknown. The membrane invaginations provide a structural scaffold for the assembly of the viral replication machinery, where the viral RNA, helicase NS3 and polymerase NS5 are associated with the cytoplasmic side of the invagination membrane, integral membrane proteins NS2A, NS2B, NS4A and NS4B impregnate the lipid bilayer and dimers of NS1 protein stabilize the replication complex from the luminal side of the ER through interactions with NS4A and NS4B (reviewed in Reference [29]). 

The virion assembly sites of the members of the Flavivirus genus, including Zika virus, are yet another virus-specific domain that forms on the ER membranes of infected cells. The virion formation sites have the capsid protein C associated with the cytoplasmic side of the ER membrane and the viral surface glycoproteins prM and E lining the luminal side of the ER membrane. The correct folding of the flavivirus glycoproteins appears to be facilitated by the host chaperones such as calreticulin, calnexin and GRP78 (also called BiP) that have been shown to interact with the envelope glycoproteins accumulating in the ER [57,58,59,60]. The viral RNA associates with the capsid C protein and a nascent virion subsequently buds into the ER lumen. Electron microscopy studies reveal a close juxtaposition of the virion assembly sites to the replication invaginations in different cell types infected with different flaviviruses, including Zika virus [43,44,45,61,62]. Such close spatial arrangement isolates the RNA replication-virion assembly interface from the cellular milieu and is likely important for the specificity of RNA incorporation into virions, since no packaging signal has been identified in the viral RNA and interaction of the C protein with RNA also does not seem to be specific [63,64]. 

The process of virion budding into the ER resembles the formation of intraluminal vesicles within endosomes to form multi-vesicular bodies in uninfected cells. This process involves the sequestration of transmembrane proteins within a patch in the endosomal membrane (which resembles that of the accumulation of the viral capsid and glycoproteins at the virion budding site on the ER), followed by the invagination of the endosomal membrane containing the sequestered proteins into the lumen. Subsequent pinching of the bud neck results in the release of small vesicles containing the proteins into the lumen of the endosome. This process is thermodynamically unfavourable and in cells is catalysed by the endosomal sorting complex required for transport (ESCRT). Four ESCRT complexes composed of different subunits exist in mammalian cells and localize predominantly to the endosomes (and to the plasma membrane in dividing cells). ESCRT-0 recognizes ubiquitylated proteins; ESCRT-I, ESCRT-II and ALIX play roles in the concentration of the proteins into a “patch” and the deformation of membranes by inducing inward curvature; and ESCRT-III constitutes the fission machinery necessary to release the nascent vesicle into the endosomal lumen. Indeed, specific elements of the ESCRT machinery seem to be hijacked for flavivirus virion biogenesis since depletion of some ESCRT components reduced the number of mature viral particles and resulted in the accumulation of incomplete ER membrane-associated virions without a significant effect on the structures of CM and vesicle packets in Dengue virus-infected cells. Specifically, the Tsg101 component of ESCRT-1 and the CHMP2/3 and CHMP4 family members of ESCRT-III were shown to be required for the efficient formation of both JEV and DENV virions [65]. Furthermore, endogenous CHMP2B and CHMP4B were detected in JEV-infected cells adjacent to viral particles, suggesting the ESCRT pathway directly participates in membrane deformation during viral particle formation [65]. 

The increased viral protein synthesis and the formation of the RNA replication and virion assembly sites in the ER are likely to trigger a stress response within the secretory pathway. ER homeostasis is sensed and maintained by multiple systems, including a family of CREB3 transcription factors highly conserved from sponges to humans. Interestingly, these transcription factors are activated only under conditions of increased secretory demand. For instance, the single Drosophila CREB3-like factor CrebA is not required to support basal secretion in cells but is absolutely essential in specialized secretory cells such as salivary glands during secretion of copious amounts of glue proteins [66,67]. In mammals, the CREB3 family is represented by five members with tissue-specific expression patterns [68]. The human CREB3L1 is the closest orthologue of Drosophila CrebA and it has been shown to be functionally analogous [67]. CREB3L1 appears to facilitate trafficking of bulky proteins such as large collagen fibrils in chondrocytes by upregulating the expression of specialized transport components necessary to adjust the secretory pathway to handle such large particulates. Since flavivirus virions are also a relatively large particulate cargo, CREB3L1-mediated upregulation of specific transport factors could be important for the adaptation of the secretory pathway in infected cells to facilitate virion trafficking. Interestingly, analysis of the published transcriptome data of flavivirus-infected cells shows that the amount of CREB3L1 mRNA is increased >8-fold in Zika- and >28-fold in Dengue-infected cells [69]. However, the possible requirement for CREB3L1 function in Zika life cycle and the mechanism of its action remain to be defined.

## 5. The Secretory Pathway in Zika Virus Infection: Leaving the ER

The nascent virions accumulated within the ER lumen are expected to engage the cellular secretory machinery to be delivered to the next secretory compartment, the Golgi complex. The molecular working of the secretory pathway has been largely deciphered using the *S. cerevisiae* yeast and the genes involved often have designation Sec, from the mutants defective in secretion. Cargo proteins leave the ER in COPII-coated vesicles that bud from ERES (Figure 2). The formation of COPII vesicles is initiated by the association of an activated (GTP-bound) form of a small GTPase Sar1 with ERES membranes, leading to the recruitment of the Sec24/Sec23 complex that forms the inner layer of the COPII coat, followed by the association of the Sec13/31 outer layer. The Sec23 stimulates the GTPase activity of Sar1, leading to its dissociation from the membranes and this stimulatory function of Sec23 is in turn enhanced by the binding of the Sec31/Sec13 layer. Thus, the basic constituents of the COPII coat bring with them the feedback mechanism limiting the expansion of this complex on the ER membrane. The recruitment of cargo proteins into the nascent vesicle is mediated by the “inner layer” Sec24 component of the COPII complex. Sec24 directly interacts with the cargo secretion signals exposed on the cytosolic side of the ER. Thus, only transmembrane proteins could have intrinsic secretion signals accessible by Sec24. The luminal cargo proteins to be recruited into COPII vesicles must interact with ER transmembrane cargo receptors that have a luminal cargo-binding domain and a cytosolic domain presenting export signal for interaction with Sec24 (reviewed in References [70,71]). Considering that flavivirus virions are strictly intraluminal, interactions with a cargo receptor are likely to be required for their active packaging into COPII carriers. The principles guiding the interactions of soluble luminal proteins with different cargo receptors are incompletely understood and it is difficult to predict which cargo receptor(s) might be utilized by the Zika virions. Importantly, all signals responsible for flavivirus virion interaction with the cellular secretory machinery are localized in the prM-E region of the polyprotein, because expression of only this fragment is sufficient for the cells to form and secrete subviral particles with structural and antigenic properties resembling those of mature virions [72,73,74,75,76]. Moreover, protein E could be secreted when it is expressed individually but the secretion increases dramatically when E is co-expressed with prM, either in cis or in trans, suggesting that a prM-E complex may present an optimized interface for interacting with the ER cargo receptor proteins [77]. Interestingly, mutations in the non-structural protein NS2 of West Nile and Yellow fever virus were shown to affect assembly and secretion of infectious virions but not empty virus-like particles containing prM-E [78,79,80]. Mutation of glycosylation sites on the E glycoprotein reduced the rate of secretion of Dengue particles due to retention in the ER, suggesting that a lectin-like intraluminal domain of a cargo receptor might be involved in virion sorting [81,82]. However, the glycosylation sites are not strictly conserved among flavivirus strains and glycosylation seems to affect flavivirus secretion only from mammalian but not from arthropod cells [83], indicating that alternative virus-receptor interactions exist. 

Moreover, the available data suggest that different flaviviruses may rely on different receptors for virion exit from the ER, and/or that they may use unconventional pathways of cargo sorting. It has been reported that the prM proteins of Dengue viruses 1–3 but not those of Dengue 4 or West Nile virus, interact with the cellular transmembrane KDEL receptor (KDELR) proteins 1 and 2 and that this interaction is required for transferring the virions from the ER to the Golgi [84]. The interaction of DENV 1–3 prM with KDELRs is mediated by three, positively charged, N-terminal amino acids on prM, H2, R19 and K21 [84]. This ER-to-Golgi transport role for KDELRs is significantly different from its function in non-infected cells, where KDELRs transfer proteins in the opposite direction, from the Golgi to the ER by interacting with cargo proteins through their C-terminal KDEL motif [85]. Thus, the Dengue virus prMs interact with KDELRs in the ER via a sequence motif that is different from the KDEL motif used in the Golgi and thereby imparts a new function to these cellular factors. 

It is currently assumed that flavivirus virions exit the ER by being incorporated into COPII vesicles but this presents a challenge for the conventional secretory pathway. The fully formed regular COPII vesicles have a diameter of ~60–100 nm, barely sufficient to fit one immature flavivirus virion of ~50 nm diameter found inside the ER lumen. Moreover, the accumulating evidence demonstrates that Zika virions associate into extensive paracrystalline lattices and/or clusters inside the ER and that at least some of these assemblages transit in their entirety all the way to the plasma membrane [44,86,87]. So how can the COPII machinery manage to transport large viral agglomerates? One possible solution to this problem is that virion clusters could engage multiple cargo receptors which in non-infected cells are associated with much smaller cargo. Exposure of an array of trafficking signals of these receptors on the cytoplasmic side of the ER may increase the nucleation area of COPII coat and lead to the formation of much bigger COPII vesicles. Another possibility is that flaviviruses engage a specialized secretory machinery normally reserved for trafficking of large cargo such as chylomicrons or collagen fibrils in specialized cells. Although these unusually large cargoes also exit the ER in COPII structures, these COPII carriers bud from ERES that are distinct from ERES that bud “normal” COPII vesicles. The formation of these modified COPII structures and sorting of unusual cargo into them requires the expression of specific isoforms of the GTPase Sar1 (Sar1b) and the cargo-interacting Sec24 (Sec24d), as well as accessory proteins such as TANGO1 and its binding partner cTAGE5 [88,89,90]. TANGO1 is a transmembrane protein that acts as a receptor for specific large cargo molecules, like collagen fibrils. cTAGE5 is a TANGO1 homologue that is also anchored in the ER membrane and has a similar cytosol-exposed part but lacks luminal cargo-interacting domain. The mechanism of how cTAGE5 and TANGO promote formation of large COPII carriers is not well understood but it seems that a concerted engagement of cTAGE5 and TANGO1 increases the recruitment of Sec12, a guanine nucleotide exchange factor for the GTPase Sar1, likely leading to more effective and perhaps sustained nucleation of COPII subunits on membranes and the consequent formation of much larger COPII-coated vesicles. Another mechanism of accommodating large cargo for export from the ER, operating during secretion of chylomicron particles from enterocytes, depends on the expression of a specific isoform of Sar1, Sar1b. Compared to the Sar1a isoform commonly expressed in mammalian cells, the GTPase activity of Sar1b is less stimulated by an interaction with the Sec13/31 complex, leading to an increased concentration of the activated GTPase on membranes and consequently causes the assembly of larger COPII structures [91]. Interestingly, the expression of cTAGE, TANGO1, Sar1b as well as the Sec24d isoform in cells secreting large cargos is under CREB3L1 transcriptional control (which is upregulated in Zika-infected cells) and these large cargo-specialized factors are also upregulated in flavivirus-infected cells [69]. 

## 6. The Secretory Pathway in Zika Virus Infection: Moving Through the Golgi? 

The dependence of infection on Golgi-resident proteins SPCA1, ERI3 and furin, the pattern of glycosylation of the flavivirus envelope glycoproteins, the importance of low pH environment characteristic of trans-Golgi for the furin-mediated cleavage of the glycoprotein prM and rearrangement of the glycoprotein E on the virion surface, all imply that the virions must transition through a Golgi-like environment on their way to the plasma membrane [92,93,94,95]. Yet, in EM images of infected cells, the well-defined stacks of the Golgi cisternae typical for non-infected cells are no longer detectable [44,86,87]. Moreover, light microscopy observations demonstrate the fragmentation of the cis and trans Golgi in infected cells [44]. In the case of the West Nile virus, a trans-Golgi marker GalT was shown to relocate and co-localize with the sites of viral RNA replication visualized by antibodies against double-stranded RNA [96]. Given that RNA replication and virion assembly sites are juxtaposed to each other in flavivirus-infected cells, these data suggest that the Golgi enzymatic machinery is likely directly recruited to the membranous compartments accumulating flavivirus virions, in fact transforming them into a new chimeric ER-Golgi-like structure. 

Cells infected with diverse arthropod-borne flaviviruses are known to secrete virions containing both furin-cleaved and non-cleaved prM glycoproteins and the level of prM maturation determines such important parameters of infection as virion interaction with antibodies and infectivity (reviewed in Reference [97]). The redistribution of the Golgi enzymatic machinery to the ER-derived compartments containing clusters of virions may account for the generation of such a mixture of virions at various degree of maturation because access to the transmembrane furin will be sterically hindered to viral particles positioned away from the membrane. 

Another indication that secretory trafficking is significantly modified in Zika virus-infected cells is the reconfiguration of the GBF1-dependent network (GBF1 stands for Golgi-specific Brefeldin A-resistant guanine nucleotide exchange factor 1). In non-infected cells, the recycling of membranes from the Golgi to the ER supports the dynamic equilibrium between these two organelles and is dependent on the activity of the GBF1 protein, a guanine nucleotide exchange factor for several GTPases of the Arf family. Arf GTPases in their activated, GTP-bound form, associate with membranes and recruit effector proteins. Human cells express five Arf isoforms, divided into 3 classes (class I: Arf1 and Arf3; class II: Arf4 and Arf5; and class III: Arf6) based on their sequence and structural homology. In uninfected cells, GBF1-activated Arfs (likely class I and II but the exact activation profile of GBF1 in vivo is unclear) recruit the COPI coatomer complex to initiate COPI vesicle formation for Golgi-to-ER recycling (Figure 2). 

Several studies have identified GBF1 as an important cellular factor for Zika virus infection, as inhibiting GBF1 enzymatic activity by small molecule inhibitors BFA or GCA is detrimental for replication of diverse flaviviruses, including Zika virus [96,98,99]. In addition, proteomics studies identified GBF1 as an interactor of some Zika proteins [98,100,101,102,103]. The GBF1-dependent Arf activation that supports COPI pathway has been implicated in the trafficking of the capsid protein C to lipid droplets, which may serve as a depot of this viral protein regulating its availability for the virion assembly process [104,105]. However, recent studies suggest that the role of GBF1 in infection could not be fully explained by GBF1 functioning solely in the assembly of COPI carriers. First, inhibition of flavivirus replication requires much higher concentrations of GBF1 inhibitors BFA and GCA than those sufficient to block the functioning of the secretory pathway in uninfected cells [98]. Second, silencing of individual Arfs or their combinations revealed that virion secretion was only moderately affected by the simultaneous depletion of Arf1 and 4 which disrupts the COPI-dependent pathway and inhibits secretion. At the same time, the simultaneous depletion of Arf4 and Arf5, which has no effect on COPI assembly or secretion in uninfected cells, practically abolished secretion of Dengue virions. The inhibition was attributed to the role of Arf4/5 in the retrieval of KDELRs hijacked by the virions to exit the ER [84,106]. These results suggest that a significant portion of flavivirus virions is delivered to the plasma membrane via GBF1-requiring but COPI-independent secretory mechanisms [44,86,87,107].

Interestingly, BFA and GCA inhibit the development of infection when added early during the infection cycle but rapidly lose their effectiveness if added after ~12 h, at the time of active virion maturation and release [96,98]. This may suggest that GBF1 is involved not only in the early steps of virion assembly by regulating the availability of the capsid protein but also may support Zika RNA replication directly, as has been described for many diverse (+)RNA viruses [108,109,110,111,112]. 

## 7. The Secretory Pathway in Zika Virus Infection: Exit from the Cell

Compared to the membrane compartments associated with the earlier events in the infectious cycle, such as RNA replication and virion assembly, the morphological structures of the final stage of infection responsible for the actual release of virions into the extracellular space have been studied in much less detail. Since Zika virions assemble and undergo maturation inside the lumen of membranous compartments, their delivery to the extracellular space is expected to proceed through fusion of a membranous carrier (containing the virion inside) with the plasma membrane. EM studies have identified large vesicles containing multiple Zika virions, as well as small vesicles containing a single virion close to or fusing with the plasma membrane [86,107]. In addition, Zika virions have been observed inside small vesicles present within bigger membrane-enclosed structures [86]. Neither the large nor the small virion-containing packets were clathrin coated, thus ruling out the clathrin-dependent sorting pathways delivering material to the plasma membrane. 

**Figure 3:**
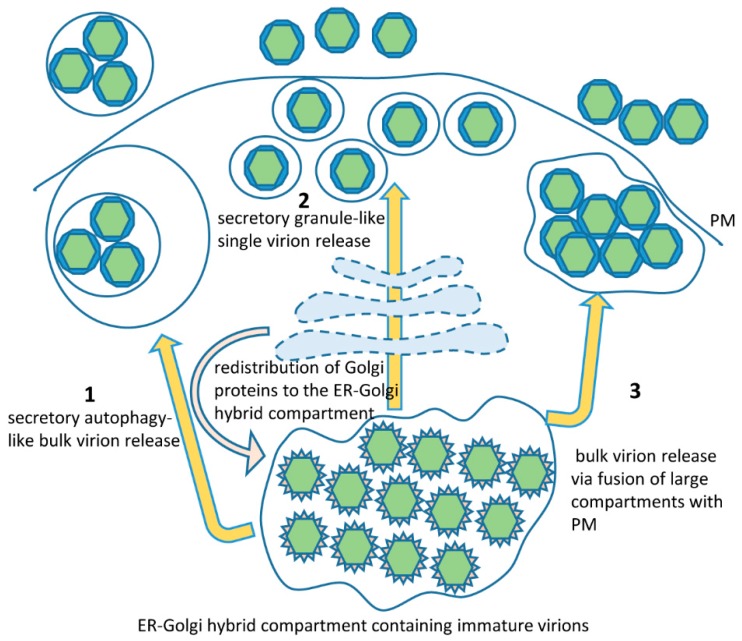
Possible Zika virion secretion pathways. Immature virions accumulate in the ER-Golgi hybrid compartment where the viral surface glycoproteins undergo final glycosylation and proteolytic maturation. Membrane-wrapped clusters of virions could be secreted through a secretory autophagy-related pathway (pathway 1). Individual virions can be released via a secretory granule-like mechanism after repackaging into individual small membranous carriers (pathway 2), which seems to be the major mechanism of virion egress. Virion clusters could be released via direct fusion of the large membranous compartments with the PM (pathway 3).

The large vesicles containing multiple mature virions are possibly the descendants of the large membranous packets generated at the ER and containing paracrystalline arrays of virions [44,86,87]. Those large vesicles would be expected to have ER-Golgi membranes around them since they would originate from a common compartment after the virus-induced redistribution of Golgi proteins to the replication membranes (Figure 3). As such, they would contain Golgi-derived molecular determinants that may allow their transport to the PM and may also have the Golgi-derived machinery to facilitate their fusion with the PM.

Multiple Zika virions have also been observed inside vesicles enclosed within bigger membrane-bound structures, perhaps suggesting that secretory autophagy could be involved in virion release [86]. Importantly, Zika virions secreted by this pathway would still be enclosed within membranous vesicles in the extracellular space and this may have significant implications for the pathogenesis of Zika-associated diseases since such virions will be protected from interactions with antibodies (Figure 3). The release of virions still enclosed in membranous vesicles may be a common feature of infection of different RNA viruses and may represent an important component of immune evasion in mammalian hosts [113,114,115,116]. 

While we are not aware of studies directly comparing the relative levels of virions secreted via fusion of large versus small vesicles with the plasma membrane, the scanning electron microscopy observations of the surface of Zika-infected Vero cells suggest that the majority of virions are released as individual particles [107]. This implies that at some point the majority of the virions present in the large ER-derived membranous structures containing multiple virions should undergo repackaging into individual membranous carriers (Figure 3). The mechanism of such repackaging is unknown but may be related to the trans-Golgi-dependent generation of secretory granules operating in some specialized cell types. Supporting the hypothesis of involvement of the secretory granule-related pathways in the final steps of flavivirus virion secretion, these scanning EM images demonstrate that Zika virion release is a massive but transient event, so that in a population of infected cells only a small proportion of them actively secrete virus at any given moment. Such a pattern is highly reminiscent of massive discharge of secretory granules, which can be observed in exocrine, endocrine, hematopoietic and neuronal cells (reviewed in Reference [117]). Furthermore, infectious Dengue virions were detected in *bona fide* secretory granules in human skin mast cells, confirming the flavivirus ability to hijack secretory granule packaging machinery at least in some cell types [118].

Although the nature of the carriers that deliver virions to the PM remains unclear, Dengue virus virion secretion has a strong dependence on the components of the EXOCYST complex, a multiprotein machinery involved in tethering secretory carriers to the plasma membrane in uninfected cells [119]. Knockdown of the EXO84 component of the EXOCYST had no effect on Dengue virus replication but significantly reduced virus secretion [119]. Expression of another EXOCYST component, EXO70, has been shown to strongly increase from 18 h post infection with Dengue virus, suggesting that late steps of infection necessitate high levels of cellular EXOCYST [119]. At the same time, while inhibition of EXOCYST function reduced virion secretion, the reduction never reached more than 75%, consistent with utilization of multiple alternative pathways of virion release [119]. Whether EXOCYST is also involved in Zika virion release and whether it performs in infected cells a function analogous to that in uninfected cells by tethering membrane carriers filled with virions to the PM remains to be determined.

Curiously, the secretion of Zika virions is strongly temperature-dependent, reaching maximum at 28 °C and decreasing at 37 °C [120]. The suboptimal virion secretion at the mammalian body temperature could be a consequence of infection-induced alterations of the cellular secretory machinery or may reflect a necessary balance the virus has to maintain to engage the secretory machinery in both mammalian and mosquito hosts. Comparative studies of Zika virus replication in mammalian and insect cells revealed that virion secretion from insect cells is much more efficient, since it took much less infected insect cells than mammalian ones to secrete the same amount of the virus to the medium [87]. The more efficient virion secretion from the insect cells could be due to the evolutionary history of mosquito-borne flaviviruses, which are believed to have evolved from obligate insect pathogens [121,122]. 

Although the basic secretory machinery is highly conserved in all eukaryotic cells, it is likely that virion traffic and release may proceed differently in different cell types in the human host body. For example, the initially infected skin fibroblasts and keratinocytes would traffic Zika virions differently than infected monocytes used by the virus to build viremia, since monocytes have a highly expanded trafficking pathway to secretory granules [123]. Thus, virion release pathways in distinct cell types may have distinct bottlenecks and vulnerable points, which may have important implications for designing anti-viral interventions. 

## 8. Concluding Remarks

Zika and related flaviviruses rely on the organelles and the biochemical networks of the cellular secretory pathway for the synthesis of all viral components, as well as the subsequent virion assembly, maturation and release. Still, our understanding of how the virus alters the compartments of the secretory pathway and repurposes the trafficking machinery that cause the massive reorganization of ER membranes to create specialized domains associated with polyprotein processing, RNA replication and virion assembly sites, and how viral infection affects the redistribution of Golgi glycosylation and proteolytic enzymes and alters lipid synthesizing and exchange machinery is very limited. Interactions of viral and cellular factors as well as activation of cellular feedback mechanisms aimed to restore secretory homeostasis likely play coordinated roles in reconfiguring the cellular secretory machinery in infected cells. The efficiency of the secretory pathway hijacking may be different in different infection-relevant cell types, depending on the availability of certain factors, which may present important opportunities to control infection. Importantly, the virus ability to adapt to interventions targeting the infection-specific modifications of the secretory pathway may be limited by the necessity to sustain secretion in different mammalian and mosquito cell types. 

## Figures and Tables

**Table 1:** Host secretory and membrane trafficking factors involved in Flavivirus life cycle.

Host Factor	Shown to Be Required for Viruses	Factor Function in Non-Infected Cells	Factor Function during Viral Replication
Furin	Multiple flaviviruses	Intraluminal protease of the TGN	Cleaves viral glycoprotein prM into the mature M
Fatty acid synthase	Dengue 2, 4, West Nile, Yellow fever	Synthesizes long chain fatty acids needed for membranes biogenesis	Recruited to convoluted membranes, potentially to generate lipids to support ER expansion; upregulated during viral infection
Reticulon 3.1A	Dengue 2, West Nile, Zika	Involved in maintaining the tubular dynamic structure of the ER	Required for the formation of viral vesicle packets
Calreticulin Calnexin GRP78	Multiple flaviviruses	ER lumen chaperones involved in protein folding	Facilitate proper folding of viral proteins; may participate in viral particle assembly
ESCRT-I	Dengue 2, Japanese encephalitis	Required for the concentration of cargoes on endosomal membranes and deformation of membranes to form lumen-facing vesicles	The Tsg101 component of ESCRT-I is required to efficiently form and bud virions into the ER lumen
CHMP2/3 CHMP4	Dengue 2, Japanese encephalitis	CHMPs are family members of ESCRT-III that facilitates fission of endosomal lumen-facing vesicles to generate multi-vesicular-bodies; this process generates exosomes	CHMPs are required to efficiently form virions. CHMP2B and CHMP4B are adjacent to viral particles in JEV-infected cells.
KDEL receptor 1 and 2	Dengue 1-3	KDELRs interact with ER-escaped proteins carrying the C-terminal KDEL motif in the Golgi and sort them into recycling COPI vesicles destined for the ER	KDELRs interact with prM to potentially assist with virion egress from the ER
ERI3	Dengue 2, Yellow fever	Golgi localized exonuclease	ERI3 relocates to sites of viral replication; has essential role in viral RNA synthesis (function unclear but ERI3 is not required for viral RNA stability or translation
SPCA1	Dengue 2, West Nile, Zika	TGN localized calcium transporter that regulates the activity of furin	Necessary for maturation of viral glycoproteins, probably through impacting furin activity
GBF1	Dengue 2, Zika	Facilitates GDP/GTP exchange to activate Arfs, which then support the recycling Golgi-to-ER COPI recycling pathway	Recruited to replication sites; function unknown
EXOCYST complex	Dengue 2	Tethers Golgi-derived secretory vesicles to the plasma membrane prior to fusion	The EXO84 component is required for optimal viral secretion but not replication; the EXO70 component is upregulated 18 h past infection
